# Innate Immune Responses Associated with Resistance against *Haemonchus contortus* in Morada Nova Sheep

**DOI:** 10.1155/2019/3562672

**Published:** 2019-11-11

**Authors:** João Henrique Barbosa Toscano, Cintia Hiromi Okino, Isabella Barbosa dos Santos, Luciana Aparecida Giraldelo, Marei Borsch von Haehling, Sérgio Novita Esteves, Ana Carolina de Souza Chagas

**Affiliations:** ^1^Faculdade de Ciências Agrárias e Veterinárias, UNESP, Via de Acesso Prof. Paulo Donato Castellane, 14884-900 Jaboticabal, São Paulo, Brazil; ^2^Embrapa Pecuária Sudeste, Rodovia Washington Luiz, Km 234, Fazenda Canchim, 339, 13560-970 São Carlos, SP, Brazil; ^3^Centro Universitário Central Paulista, Rua Miguel Petroni, no. 5111, 13563-470 São Carlos, São Paulo, Brazil

## Abstract

The immune response against *Haemonchus contortus* infections is primarily associated with the Th2 profile. However, the exact mechanisms associated with increased sheep resistance against this parasite remains poorly elucidated. The present study is aimed at evaluating mediators from the innate immune response in lambs of the Morada Nova Brazilian breed with contrasting *H*. *contortus* resistance phenotypes. Briefly, 287 lambs were characterized through fecal egg counts (FEC) and packed cell volume (PCV) after two independent experimental parasitic challenges with 4,000 H. *contortus* L_3_. 20 extreme resistance phenotypes (10 most resistant and 10 most susceptible) were selected, subjected to a third artificial infection with 4,000 L_3_, and euthanized 7 days later. Tissue samples were collected from abomasal fundic and pyloric mucosa and abomasal lymph nodes. Blood samples were collected at days 0 and 7 of the third parasitic challenge. RNA was extracted from tissue and blood samples for relative quantification of innate immune-related genes by RT-qPCR. For the abomasal fundic mucosa, increased *TNFα* and *IL1β* expression levels (*P* < 0.05) were found in the susceptible animals, while resistant animals had *IL33* superiorly expressed (*P* < 0.05). Higher levels (*P* < 0.05) of *TLR2* and *CFI* were found in the abomasal pyloric mucosa of resistant animals. *TNFα* was at higher levels (*P* < 0.05) in the blood of susceptible lambs, at day 0 of the third artificial infection. The exacerbated proinflammatory response observed in susceptible animals, at both local and systemic levels, may be a consequence of high *H*. *contortus* parasitism. This hypothesis is corroborated by the higher blood levels of *TNFα* before the onset of infection, which probably remained elevated from the previous parasitic challenges. On the other hand, resistant lambs had an enhanced response mediated by TLR recognition and complement activation. Nevertheless, this is the first study to directly associate sheep parasitic resistance with IL33, an innate trigger of the Th2-polarized response.

## 1. Introduction

*Haemonchus contortus* infections are the main cause of economic losses to sheep farming in tropical countries. This gastrointestinal nematode (GIN) is considered the most pathogenic sheep parasite, and it is the prevalent species in most of the Brazilian territory [1–4]. The losses are due to decreased productivity, sheep mortality, and expenses with anthelmintic treatments [1, 5]. The inadequate use of anthelmintics led to a widespread multiple resistance against most of the commercially available molecules [6–9], which highlights the importance of alternative control methods, such as selection of genetically resistant animals, and the development of immunotherapeutic or imunoprophylactic tools. Therefore, it is essential to understand the genetic or immune-related mechanisms involved in the development of host resistance against GIN infections.

The immune response of sheep against GIN infections is primarily associated with the adaptive Th2-polarized profile, with local release of the interleukins IL4, IL5, and IL13, in addition to IgE production, eosinophilia, and mastocytosis [10–13]. However, the exact mechanisms associated with increased sheep resistance against *H*. *contortus* infections remains poorly elucidated, especially regarding the involvement of the innate immunity.

The activation of Toll-like receptor (TLR) genes (especially *TLR2*, *TLR4*, and *TLR10*) has been associated with host defense against *H*. *contortus* [14, 15]. In addition, the activation of the nuclear factor *κ*B (NF*κ*B) pathway induces inflammatory response in the early stages of parasitic infection, with increased production of proinflammatory cytokines, such as TNF*α* and IL-1*β* [16–18]. In resistant animals, this response is rapidly replaced by the induction of anti-inflammatory activity, with increased levels of IL10 and TGF*β* [14, 19]. On the other hand, susceptible animals present a persistent inflammatory response, with a high expression of NF*κ*B signaling pathway molecules (*IKBKB* and *NFKBIA*) and proinflammatory cytokines (*IL1β*, *IL6*, and *TNFα*), followed by a late expression of regulatory markers (*IL10* and *TGFβ*) [14].

The reactive oxygen species, as nitric oxide, are well known for its antimicrobial activity and are associated with cytotoxic effect against GIN [20]. This molecule is stimulated by inducible nitric oxide synthase (iNOS) released by activated effector cells. In murine models, both iNOS and nitric oxide were proved to be involved in resistance against parasitic nematodes [21]. The activation of genes responsible for producing reactive oxygen species (*NOS2A*) was directly associated with increased resistance of sheep against *H*. *contortus* and *Trichostrongylus colubriformis* [14].

GIN infection leads to the activation of the alternative pathway of the complement system [22, 23], and the action of the resulting opsonins has been proved to be lethal to GIN larvae [24]. This pathway involves the spontaneous cleavage of C3 into active forms, C3a and C3b, with strong opsonizing properties. Besides, like the other pathways, alternative activation of the complement results in the formation of the terminal complex (C5-C9) [25]. Although, due to the high abundance of C3 at mucosal surfaces, regulatory mechanisms are required to avoid hyperactivation of this pathway, in which complement factor I (CFI) plays an essential role [26]. Superior activation of genes directly associated with complement activation (*C7* and *CFI*) has been observed in sheep resistant to *H*. *contortus* [27].

Recent studies have shown the importance of interleukins IL25 and IL33 in the early phase of defense against GIN [28–30]. These “alarmins” are constitutively expressed in epithelial cells of the mucosal barriers, the first cells to have contact with the invading pathogens. In response to tissue injury, there is a release of IL25 and IL33 [31], potent inducers and enhancers of Th2 profile immune response, by stimulating type 2 innate lymphoid cells (ILC2) and CD4+Th2-polarized cells [31–33]. As for sheep, *Trichostrongylus colubriformis* infection was previously associated with upregulation of *IL33* in the intestinal mucosa [34]. However, regarding the role of IL25, there are no previous studies evaluating this cytokine in GIN-infected sheep.

The Morada Nova, a Brazilian hair sheep breed, is well known for its improved natural resistance against GIN infections compared with other breeds such as Dorper, Texel, Ile de France, and Santa Inês [35–37]. Most of studies have compared immune profiles between sheep breeds with different levels of parasitic resistance, while studies targeting immune responses inside breeds are scarce and absent for the Morada Nova breed. Therefore, the present study is aimed at evaluating innate immune mediators in the abomasum and abomasal lymph nodes of Morada Nova lambs with opposing resistance phenotypes against *H*. *contortus* infections. Furthermore, the systemic inflammatory profile was also assessed in the blood of these animals. Nevertheless, this is the first study to investigate the role of the “alarmins” in the *H*. *contortus* resistance.

## 2. Materials and Methods

### 2.1. Experimental Lambs and Animal Management

287 Morada Nova lambs, 146 males and 141 females, were weaned at approximately a hundred days of age. The lambs were kept with their mothers in 3 hectares of pasture covered with Aruana grass (*Panicum maximum* cv. Aruana) and fed in a creep feeding system until weaning. Newly weaned lambs were allocated to four paddocks having the same pasture composition described above and were separated by sex. In the summer (rainy season), they were fed exclusively at the pasture. During dry season, they were supplemented with corn or grass silage added with pelleted citrus pulp. Water and mineral salt were supplied *ad libitum* throughout the experiment.

### 2.2. Phenotyping for *Haemonchus contortus* Resistance

At weaning, lambs were naturally infected with GIN, with a mean fecal egg count (FEC) of 6,643 ± 8,994 eggs per gram of feces (EPG). *Haemonchus* (96.4%) was the predominant genus in fecal cultures, followed by *Cooperia* (2.1%) and *Trichostrongylus* (1.5%). To eliminate the natural infection, all animals were dewormed with monepantel (Zolvix®, Novartis Animal Health, Brazil) at a 2.5 mg/kg dose. Nematode-free status was confirmed after two negative FECs (days 7 and 14 post-treatment). 15 days after deworming, the lambs were experimentally infected with a single oral dose of 4,000 H. *contortus* L_3_ (day zero: D0). In this occasion, blood samples were collected for packed cell volume (PCV) determination. Individual FECs were performed every seven days from D21, and PCV was determined every fourteen days from D14. On D42 of the first parasitic challenge, the lambs were dewormed another time and, 15 days later, submitted to a second parasitic challenge, following the same chronogram previously described.

The lambs were classified according to their parasitic resistance level based on the averages of FEC and PCV after artificial infection (excluding post-deworming values: D0). Among these, the 10 most resistant (lowest FEC and highest PCV) and the 10 most susceptible (highest FEC and lowest PCV) were identified. These animals were dewormed on D42 of the second parasitic challenge and placed in previously decontaminated cemented stalls, in order to avoid natural GIN infections. After 15 days, they were once again infected with 4,000 H. *contortus* L_3_ and euthanized seven days later. Necropsy was performed and tissue samples were collected from the abomasal mucosa (fundic and pyloric regions) and abomasal lymph nodes, which were immediately snap frozen in liquid nitrogen (-196°C) and stored at -80°C until processing. Blood samples were collected in PAXgene Blood RNA tubes (Preanalytix, Valencia, USA) at D0 and D7 of the third parasitic challenge. These samples were kept at room temperature (25°C) for 12 h and then frozen at -20°C until process.

### 2.3. Target Gene Selection and Primer Design

A total of 15 target genes related to the innate immune responses were selected for relative quantification by reverse transcription followed by real-time quantitative PCR (RT-qPCR): pattern recognition receptors (*TLR2*, *TLR4*, *TLR7*, and *TLR10*); molecules of the NF*κ*B signaling pathway (*NFKBIA*, *IKBKB*); inducible nitric oxide synthase (*NOS2A*), alarmin cytokines (*IL25* and *IL33*); proinflammatory cytokines (*TNFα* and *IL1β*); anti-inflammatory cytokines (*TGFβ* and *IL10*); and complement system components (*C7* and *CFI*). All target genes were quantified in the tissue samples, whereas, only the proinflammatory cytokines *TNFα* and *IL1β* were evaluated in blood samples, due to the lower yield of RNA extraction.

All primer pairs were designed using Primer3 (http://primer3.ut.ee/), based on messenger RNA (mRNA) sequences deposited in Genbank (https://www.ncbi.nlm.nih.gov/genbank/) and gene sequences from Ensembl (http://www.ensembl.org/index.html). Whenever possible, primer spanning at least one junction between two adjacent exons were selected in order to avoid genomic DNA amplification. Primer sequences were analyzed by Netprimer (http://www.premierbiosoft.com/NetPrimer/AnalyzePrimerServlet) and Oligoanalizer (https://www.idtdna.com/calc/analyzer), to avoid secondary structures formation. The specificity of primer pairs was verified by aligning the sequences with those deposited on international databases, using the Basic Local Alignment Search Tool (BLAST, https://blast.ncbi.nlm.nih.gov/Blast.cgi).

### 2.4. RNA Extraction and Complementary DNA (cDNA) Synthesis

Total RNA was extracted from tissue samples using QIAzol® Lysis Reagent (Qiagen) and Tissue Ruptor (Qiagen), followed by RNA purification in silica columns using RNeasy Mini Kit (Qiagen). Blood samples collected in PAXgene tubes were submitted to RNA extraction using the PAXgene Blood RNA Kit (Preanalytix), following the manufacturer's recommendations. The concentration and purity of RNA samples were estimated in the spectrophotometer NanoDrop™ 2000 (Thermo Scientific, Cleveland, USA), by absorbance readings at 260 nm (A_260_) and A_260_/A_280_ ratios, respectively. The RNA integrity was confirmed by 1.0% agarose gel electrophoresis.

Complementary DNA (cDNA) synthesis was performed using 1,500 ng of total RNA, High-Capacity cDNA Reverse Transcription Kit (Applied Biosystems, Foster City, USA), and oligo(dT) primers (IDT) in T100™ Thermal Cycler (Bio-Rad, Redmond, USA). For the amplification of intronless genes (*TLR2* and *TLR10*) and *TLR7* (primers designed out of exon-exon junction), RNA samples were treated with DNAse I (Thermo Fisher) before reverse transcription (RT).

### 2.5. Real-Time Quantitative PCR (qPCR)

Real-time quantitative PCR (qPCR) was performed based on SYBR Green I DNA intercalating dye system, using Quantifast SYBR Green PCR kit (Qiagen), in MicroAmp® Optical 96-Well Reaction Plates (Applied Biosystems) and sealed with MicroAmp® Optical Adhesive Film (Applied Biosystems). The reaction mix consisted of 7.5 *μ*L of 2X Quantifast SYBR Green PCR Master Mix, 0.3 *μ*L each primer at 10 *μ*M concentration, 1.9 *μ*L of ultrapure water, and 5 *μ*L of cDNA at 4 ng/*μ*L concentration (20 ng per reaction), in a final volume of 15 *μ*L. The qPCR assays were carried out in a 7500 Real-Time PCR System Thermal Cycler (Applied Biosystems), and cycling conditions consisted of preincubation at 95°C for five minutes, 40 cycles of denaturation at 95°C for 15 seconds, and annealing/extension at 60°C for 35 seconds, followed by melting curve analysis with temperature ranging between 65°C and 95°C, at 0.5°C increments every five seconds. All samples were tested in duplicate. No-template controls (NTC) were included for each qPCR run.

In addition, the amplified products were subjected to 2.5% agarose gel electrophoresis to confirm the expected fragment sizes. The efficiencies of the optimized qPCR reactions were determined from linear regression by plotting Cq values and respective cDNA concentration from seven five-fold serial dilutions of cDNA (75 ng/*μ*L).

The relative gene expression was normalized by the reference gene *YWHAZ* (abomasal pyloric mucosa) and by the geometric mean of the Cq values of *GAPDH* and *YWHAZ* (abomasal fundic mucosa and abomasal lymph nodes), according to the stability test previously performed [38]. For the blood samples, five different genes (*GAPDH*, *G6PDH*, *YWHAZ*, *ACTB*, and *B2M*) were tested with the programs BestKeeper, NormFinder, and RefFinder, which identified *YWHAZ* as the most stable reference gene. The expression levels were calculated according to Pfaffl [39]. For each gene/tissue combination, the sample with the lowest expression level (highest *Δ*Cq) was used as a calibrator.

### 2.6. Statistical Analyses

The relative gene expression levels for the different target genes between the extremes of infection by *H*. *contortus* in the abomasal mucosa and respective lymph nodes were compared by the Mann-Whitney *U* test. Gene expression levels of systemic inflammatory responses were compared with a generalized linear model (GLM) with repeated measures of the same animal, considering the effects of phenotypic group, collection date, and interaction. Statistical analyses were performed with the software RStudio (version 1.1.463), at a 5% significance level. All the graphs were plotted using GraphPad Prism (version 7.0a).

## 3. Results

### 3.1. Selection of the Extreme Resistance Phenotypes

20 animals classified as extreme resistance phenotypes (10 most resistant and 10 most susceptible) were selected. The overall mean of FEC of the two parasitic challenges for resistant lambs was quite low (192.2 ± 48.85 EPG) and significantly lower (*P* < 0.001) than that of susceptible lambs (14,981 ± 1,938 EPG). There was also a significant difference (*P* < 0.001) for PCV between resistant (36.77 ± 0.68%) and susceptible (29.25 ± 0.75%) lambs.

### 3.2. Specificity and Efficiency of the Designed Primers

All primer pairs were specific. Electrophoresis confirmed the amplification of single products of the expected sizes, and a single peak was observed at melting curve analysis. The efficiencies of the qPCR assays ranged from 92.421% to 102.740%, and the correlation coefficients (*R*^2^) ranged between 0.964 and 0.999. Primer's sequences and other information are shown in Table 1.

### 3.3. Relative Quantification of Genes Associated with Innate Immune Responses

#### 3.3.1. Local Immune Responses (Tissue Samples)

**Figure 1:**
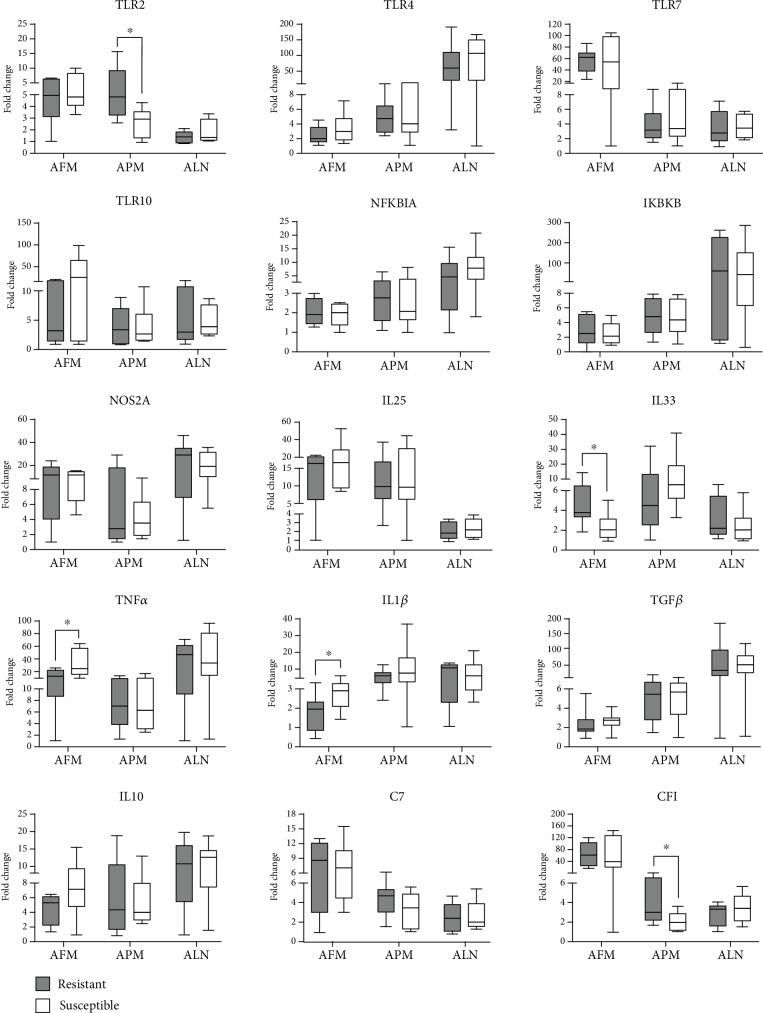


The comparison of gene expression levels of the immune-related mediators in the different tissues between the groups are represented in Figure 1. *TLR2* and *CFI* were superiorly expressed (*P* < 0.05) in the abomasal pyloric mucosa of the resistant animals, while *IL33* was at higher levels in the same group (*P* < 0.05), but in the abomasal fundic mucosa. The susceptible group, in turn, presented an exacerbated inflammation of the abomasal mucosa, represented by superior expression of both *TNFα* and *IL1β* (*P* < 0.05) in the abomasal fundic mucosa. No significant differences (*P* > 0.05) were observed for the other tested genes in any of the evaluated tissues.

#### 3.3.2. Systemic Immune Responses (Blood Samples)

**Figure 2:**
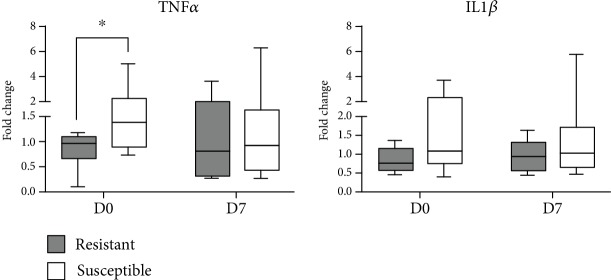


Expression levels of the proinflammatory cytokines (*TNFα* and *IL1β*) between the groups in the blood samples are shown in Figure 2. A significant difference was observed only for *TNFα*, which was found superiorly expressed (*P* < 0.05) in the susceptible animals, at D0-3. *IL1β*, in turn, did not differ (*P* > 0.05) between groups. No significant differences were observed between experimental dates (*P* > 0.05), nor significant interaction with the phenotypic group (*P* > 0.05).

## 4. Discussion

The present study evaluated gene expression levels of the innate immune response mediators in Morada Nova lambs with contrasting *H*. *contortus* resistance phenotypes. Susceptible lambs had increased levels of the proinflammatory cytokines at both local (abomasal mucosa and lymph nodes) and systemic levels (blood). Resistant animals, on the other hand, presented an enhanced local immune response mediated by TLR recognition, IL33 synthesis, and complement activation.

TLR recognition is the main mechanism of identification of invading agents by the sentinel cells [40], and it was associated with effective responses against helminths [41–43]. Ingham et al. [14] found higher levels of *TLR2*, *TLR4*, *TLR7*, and *TLR10* transcripts in the abomasal mucosa of sheep with increased resistance to *H*. *contortus* infections. In the present study, we found similar results, and *TLR2* was at higher levels in the abomasal pyloric region of the resistant lambs, which suggests an enhanced sensitivity on the recognition of the invading parasites, resulting in earlier effective immune response.

NF*κ*B is the main signaling pathway involved in the inflammatory response induced by TLR recognition [44]. Ingham et al. [14] observed that susceptible sheep had increased levels of *NFKBIA* and *IKBKB* transcripts in the jejunal mucosa three days after artificial infection with *T*. *colubriformis*. The authors hypothesized that increased levels of these transcripts could be associated with the lower *TLR* expression, resulting in lower sensitivity in parasite recognition and consequent delayed induction of the initial inflammatory response. However, in our study, no significant differences in the *NFKBIA* and *IKBKB* transcript levels were observed between groups. This difference may be due to the later time of sampling performed in our experiment, at the 7th dpi.

The reactive oxygen species, well-known antimicrobials, were previously associated with lethal effects against nematodes, including *H*. *contortus* [20, 45, 46]. The iNOS induction of NO synthesis was associated with defense against parasitic infections [21]. Higher levels of *NOS2A* transcripts were found in the abomasal mucosa of sheep with increased resistance to *H*. *contortus* [14, 47]. On the other hand, *NOS2A* knocked out mice were not affected on their resistance against GIN infection [48]. In the present study, it was not possible to associate a differential profile for *NOS2A* mRNA levels with resistance to *H*. *contortus* infection.

IL25 and IL33 are constitutively expressed cytokines, released by epithelial cells from mucosal barriers in response to cellular damage. These alarmins are known to be natural triggers and enhancers of the Th2 type immune response [28, 32, 49, 50], resulting in local and systemic induction of IL4, IL5, and IL13 synthesis, eosinophilia, and high levels of IgE [51–53]. Depletion of IL25 or IL33 was proved to abrogate natural resistance to several helminth species [29, 54–59]. Regarding sheep resistance against GIN infections, this is the first study, to our knowledge, to investigate the involvement of IL25, although no differential expression profile was observed between resistant and susceptible lambs. However, the importance of this alarmin in sheep resistance against parasitic infections cannot be ruled out, since this cytokine may be activated at an earlier stage of infection.

The involvement of IL33 in sheep response to GIN was recently evidenced. Andronicos et al. [34] and Corvan et al. [60] cultured human epithelial cells or ovine intestinal epithelium with infecting *T*. *colubriformis* larvae. Cellular necrosis induced by parasitism or stimulation with excretory and secretory products from larvae was associated with a marked increase in *IL33* transcript levels. Also, a 15-fold increase in the *IL33* expression was observed in the small intestine mucosa of sheep artificially infected with *T*. *colubriformis*, at 14th dpi [34]. The present study evidenced, at the 7th dpi, superior expression of *IL33* in the abomasal fundic mucosa of the resistant lambs. This is the first study to associate higher levels of this cytokine with sheep resistance against GIN infections. In addition, this difference between the extremes of resistance was earlier detected when compared to the findings observed by Andronicos et al. [34]. We hypothesized that resistant sheep are able to respond in an early and enhanced manner for alarmin releasing after infection.

During GIN infections, the lesion resulting from the parasitism causes inflammation of the gut mucosa, with consequence release of proinflammatory cytokines, such as TNF*α* and IL1*β*, at both local [16, 18, 61] and systemic levels [62]. However, in sheep that develop an effective response against these parasites, this initial inflammatory response is followed by induction of regulatory activity, characterized by increased expression levels of *IL10* and *TGFβ* [16, 19, 63]. Susceptible sheep, on the other hand, usually present higher mRNA levels of *TNFα* and *IL1β* in the gut mucosa and respective lymph nodes, at the early [17, 19] and late infection [12]. When comparing the kinetics of this cytokines, it was shown that susceptible sheep have a delayed and prolonged proinflammatory activity, compared to resistant animals [19]. In the present study, the results were consistent with previous reports, with higher expression levels of the proinflammatory cytokines *TNFα* and *IL1β* in the abomasal mucosa of susceptible lambs. Even, higher levels of TNF*α* were also found in the blood of lambs from this group. The pronounced inflammatory activity is probably due to an incomplete defense against parasites at the onset of infection. Thus, a larger number of surviving helminths continue to cause lesions in the gastrointestinal mucosa, leading to enhanced local inflammatory process. Nevertheless, the exacerbated systemic inflammation before the onset of infection may be a consequence of residual response induced by previous two parasitic challenges performed in these animals.

Activation of the complement system by the alternative pathway is one of the first immune events following GIN infection, resulting in opsonin activation and the formation of the terminal complex [22, 23, 64, 65]. The opsonization of the parasite surface with complement proteins plays an important role in the destruction of larval forms at the beginning of infection, due to eosinophils attraction and stimulation [24, 65, 66]. The alternative complement pathway involves a “tick over” process leading to spontaneous cleavage of C3 into its active forms, C3a and C3b [67]. The complement factor I (CFI) is the main regulatory component involved on the alternative pathway, inactivating C3 convertase and avoiding excessive production of C3b, given the abundance of C3 in plasma and mucous membranes [26, 67]. In the present study, two complement factor genes were evaluated: *CFI*, related to the control of alternative route activation and *C7*, a membrane attack complex (MAC) protein. Higher levels of *CFI* transcripts were found in the abomasal pyloric mucosa of the resistant lambs, which indicates the participation of this mediator in the initial response against *H*. *contortus* larval forms. Recent studies have also demonstrated superior expression of *C7* and *CFI* in sheep with increased resistance to *H*. *contortus* infection, at the early [68] or late infection [27].

## 5. Conclusion

Susceptible lambs had increased transcripts levels of proinflammatory cytokines *TNFα* and *IL1β*. This exacerbated inflammatory response, both locally (abomasal fundic mucosa) and systemically (blood), may be a consequence of the higher *H*. *contortus* parasitism in this group. Resistant animals, on the other hand, presented an enhanced local (abomasum) immune response mediated by TLR recognition, IL33 synthesis, and complement activation. Nevertheless, this is the first study to directly associate sheep resistance against *H*. *contortus* with higher levels of *IL33*, an innate inducer of the Th2-polarized response.

## Figures and Tables

**Table 1:** Sequences of the primers used for relative gene quantification (RT-qPCR) in Morada Nova lambs resistant or susceptible to *Haemonchus contortus* infection, including: accession number, amplicon size, exon boundary covered, efficiency (*E*), determination coefficient (*R*^2^), and slope.

Gene	Access number	Sequence (5′-3′)	Amplicon size (bp)	Exon boundary	*E* (%)	*R* ^2^	Slope
GAPDH	NM_001190390.1	F: CAAGCTCATTTCCTGGTACGAC R: TCTCTCTTCCTCTCGTGCTCCT	131	10/11	99.136	0.999	-3.343

YWHAZ	NM_001267887.1	F: CTGAGAAAGCCTGCTCTCTTGC R: GGTATCCGATGTCCACAATGTC	143	5/6	102.340	0.999	-3.267

TLR2	NM_001048231.1	F: CTCCCACTTCCGTCTCTTTGAT R: CTCCAGGTAGGTCCTGGTGTTC	133	Intronless	96.378	0.999	-3.412

TLR4	NM_001135930	F: ACCCTTGCGTACAGGTTGTTC R: ATGGCTGCCTAAATGTCTCAGG	137	1/2	100.710	0.964	-3.300

TLR7	NM_001135059.1	F: TTGAGAAGCCCCTTCAGAAGTC R: TCAGACACTGCCAGAAGTACGG	117	None	101.823	0.994	-3.279

TLR10	NM_001135925.1	F: GTGGTTATCATGCTCGTTCTGG R: TCTTCCTAACCCTGAGCCATGT	118	Intronless	95.298	0.992	-3.440

NFKBIA	NM_001166184.1	F: CGAGACTTTCGAGGAAATACCC R:GACACGTGTGGCCATTGTAGTT	141	3/4	95.848	0.999	-3.420

IKBKB	XM_015104530.1	F: AGGCTGCCGAGAAGAGTGAC R: CAAACTCTGGTCCTGCTCCTTC	104	23/24	92.421	0.986	-3.518

iNOS	AF223942.1	F: CACCTCTACTGGGAGGAGATGC R: GAACATAGACCTTGGGCTGGTC	102	24/25	99.905	0.999	-3.324

IL1*β*	NM_001009465.2	F: AGTGGTGTTCTGCATGAGCTTC R: CAGGGTCGGTGTATCACCTTTT	124	4/5	100.440	0.997	-3.311

TNF*α*	NM_001024860	F: CTCAGGTCATCTTCTCAAGCCT R: GAGGGCATTGGCATACGAG	108	2/3	94.086	0.983	-3.472

IL10	NM_001009327.1	F: CTTTAAGGGTTACCTGGGTTGC R: TCACGTGCTCCTTGATGTCAG	110	2/3	102.305	0.995	-3.268

TGF*β*	NM_001009400	F: CAGCTCCACAGAAAAGAACTGC R: GTGTCCAGGCTCCAGATGTAGG	144	5/6	98.993	0.994	-3.346

C7	XM_012096998.2	F: CTATGAATGTGGGTCCTCCTTG R: CTCCCTACCAGCCACAGTGTAA	130	16/17	102.74	0.990	-3.258

CFI	XM_004009622.3	F: ATGGAGTGTGCAGGTACAGATG R: CTCACAATACCCCAAACGTAAG	113	14/15	99.818	0.997	-3.326

## Data Availability

Previously reported reference gene stability test data were used to support this study and are available at 10.1007/s11033-018-4281-x. These prior study is cited at relevant places within the text as reference [38]. All remaining data are included within the article.
